# p16^INK4A^ Positively Regulates p21^WAF1^ Expression by suppressing AUF1-dependent mRNA decay

**DOI:** 10.1371/journal.pone.0070133

**Published:** 2013-07-23

**Authors:** Huda H. Al-Khalaf, Abdelilah Aboussekhra

**Affiliations:** 1 Department of Molecular Oncology, King Faisal Specialist Hospital and Research Center, Riyadh, Saudi Arabia,; 2 The Joint Center for Genomics Research, King Abdulaziz City for Science and Technology, Riyadh, KSA; German Cancer Research Center, Germany

## Abstract

**Background:**

p16^INK4a^ and p21^WAF1^ are two independent cyclin-dependent kinase inhibitors encoded by the *CDKN2A* and *CDKN1A* genes, respectively. p16^INK4a^ and p21^WAF1^ are similarly involved in various anti-cancer processes, including the regulation of the critical G1 to S phase transition of the cell cycle, senescence and apoptosis. Therefore, we sought to elucidate the molecular mechanisms underlying the link between these two important tumor suppressor proteins.

**Methodology/Principal Findings:**

We have shown here that the p16^INK4a^ protein positively controls the expression of p21^WAF1^ in both human and mouse cells. p16^INK4a^ stabilizes the *CDKN1A* mRNA through negative regulation of the mRNA decay-promoting AUF1 protein. Immunoprecipitation of AUF1-associated RNAs followed by quantitative RT-PCR indicated that endogenous AUF1 binds to the *CDKN1A* mRNA in a p16^INK4A^-dependent manner. Furthermore, while *AUF1* down-regulation increased the expression level of the *CDKN1A* mRNA, the concurrent knockdown of *AUF1* and *CDKN2A*, using specific silencing RNAs, restored the normal expression of the gene. Moreover, we used EGFP reporter fused to the *CDKN2A* AU-rich element (ARE) to demonstrate that p16^INK4A^ regulation of the *CDKN1A* mRNA is AUF1- and ARE-dependent. Furthermore, ectopic expression of p16^INK4A^ in p16^INK4A^-deficient breast epithelial MCF-10A cells significantly increased the level of p21^WAF1^, with no effect on cell proliferation. In addition, we have shown direct correlation between p16^INK4a^ and p21^WAF1^ levels in various cancer cell lines.

**Conclusion/Significance:**

These findings show that p16^INK4a^ stabilizes the *CDKN1A* mRNA in an AUF1-dependent manner, and further confirm the presence of a direct link between the 2 important cancer-related pathways, pRB/p16^INK4A^ and p14^ARF^/p53/p21^WAF1^.

## Introduction

Cell proliferation is activated by cyclins and cyclin-dependent kinases (CDKs), and is inhibited in response to various stresses by cyclin-dependent kinase inhibitors (CDKI) [1,2]. There are two families of CDKI, the Cip/Kip family, including p21^WAF1^ (hereafter referred to as p21), which inhibits mainly cyclin E-CDK2 complexes, and the INK family, including p16^INK4A^ (hereafter referred to as p16), which targets preferentially cyclin D-CDK4/6 [3,4].

p21 is a universal cyclin-dependent kinase inhibitor, which is able to interact and inhibit most cyclins/CDKs [5]. cDNA microarray experiments showed that increased p21 expression selectively inhibits different genes involved in cell proliferation and repair, while at the same time up-regulates multiple genes that have been associated with senescence and ageing related diseases [6]. The effects of p21 knockout in mice and its expression patterns in human cancer are consistent with a role for p21 as both tumor suppressor and also oncogene in some cell types [7–9]. Indeed, loss of p21 delayed the development of thymic lymphomas induced either by ataxia-telangiectasia mutated deficiency or by ionizing irradiation [10]. Furthermore, De la Cueva et al. have shown that the absence of p21 results in a significant extension of the lifespan of p53-null and p53-haploinsufficient mice, and a decrease in the incidence of spontaneous thymic lymphomas [11].

p16 plays important roles in tumor suppression [5,12–14]. The p16 coding-gene has been found homozygously deleted, mutated or transcriptionally inhibited by methylation in a large number of different human tumor types [3,13–15]. Mice lacking p16 are tumor prone and develop different types of cancer, particularly after exposure to carcinogens [16,17].

p16 and p21 belong to two cancer-related pathways: pRB and p53, respectively, which are inactivated in virtually all tumors [18]. Recent lines of evidence revealed the existence of functional interactions between these two important tumor suppressor proteins [19]. Indeed, both are involved in cell cycle control and both interact with CDK4 [4,5], indicating that they compete for binding this kinase [20]. Moreover, they are both suppressors of UV-induced apoptosis [21,22] and are up-regulated during aging and senescence [7,23,24]. Furthermore, beside their tumor suppressive function, p16 and p21 have also cell non-autonomous tumor-suppressive activities [25,26].

The expression of p16 and p21 is under the control of different activators and suppressors that regulate both proteins with different mechanisms. The expression of both *CDKN1A* and *CDKN2A* genes is regulated at transcriptional, post-transcriptional and post-translational levels [27–29], but they are both post-transcriptionally regulated by the AUF1 protein. Indeed, the RNA decay promoting AUF1 protein binds to the 3’ UTR of the *CDKN1A* and *CDKN2A* mRNAs and reduces their stability [30,31]. All these similarities between p16 and p21 suggest the presence of direct or indirect interaction/regulation between these 2 key cell proliferation regulators.

In the present report, we present evidence that p16 positively controls p21 expression in both human and mouse cells. This effect is mediated through p16-dependent stabilization of the *CDKN1A* mRNA through negative regulation of the RNA decay promoting AUF1 protein.

## Materials and Methods

### Cell lines, cell culture and chemicals

U2OS, EH1 and EH2 [32] (The three cell lines are a generous gift from Dr. G. Peters), MEFs p16 (WT) and their p16-specific knockout counterpart [16] and HFSN1 (primary normal human skin fibroblast) [33]. These cells were routinely cultured in DMEM/F12 medium supplemented with 10% FBS. MCF-10A cells were purchased from ATCC and were cultured according to the manufacturer recommendations. Actinomycin D and IPTG were purchased from Sigma, USA.

### Cellular lysate preparation and immunoblotting

This has been performed as previously described [32]. Antibodies directed against p21 (F-5), p14 (C-18), GAPDH (FL-335), PCNA (PC-10) and β-actin (C-11) were purchased from Santa Cruz (Santa Cruz, CA); p16 from BD Biosciences (San Jose, CA) and AUF1 from Abcam (Cambridge, MA).

### Ki-67 immunostaining

The proportion of S phase cells was determined by assessing the level of Ki-67 by immunostaining as previously described [34]. Cells were fixed in 1:1 acetone: methanol, and a standard indirect immunoperoxidase procedure was applied using Ki-67 antibody (Abcam), followed by peroxidase-conjugated anti-rabbit antibody (Dako). Sites of antibody binding were visualized by the deposition of brown polymer of DAB chromogen (Novocastra Laboratories Ltd, IL, USA). The percentage of Ki-67-labelled cells was determined for at least 500 cells per data point and expressed as a mean and standard error of triplicate determinations.

### Cell proliferation

MCF-10A cells (2x10^4^) expressing either *CDKN2A*-ORF or control plasmid were seeded in E-16 plate and the proliferation rate was measured using the RTCA-DP xCELLigence System (Roche-Germany).

### Flow cytometry

Cells were harvested and resuspended in 1 mL of PBS before being fixed by drop wise addition of 3ml of 100% methanol. Fixed cells were centrifuged, resuspended in 50 ml of RNase (1 mg/mL) and incubated for 30 min at room temperature, followed by addition of 1ml of 0.1 mg/ml of propidium iodide. Cells were analyzed for DNA content by flow cytometry (Becton Dickinson). The percentage of cells in various cell-cycle phases was determined by using Cell Quest software (Becton Dickinson).

### RNA purification, RT-PCR and real time RT-PCR

Total RNA was purified using the TRI reagent (Sigma) according to the manufacturer’s instructions. The RNA concentrations were determined using NanoDrop® ND-1000 Spectrophotometer (Nanodrop Inc., Wilmington, DE, USA). Single stranded complementary DNA (cDNA) was obtained by reverse transcription using 1µg of RNA and the RT-PCR kit (BD Biosciences) following the manufacturer’s protocol. cDNA was then amplified with 1U Taq polymerase, dNTPs (50 mM), and primers (25 pmol each). The mixture was first heated at 94°C for 5 min and then 30 cycles at 94°C for 1 min, 55°C for 1 min and 72°C for 1 min, then 72°C for 10 min. PCR products were seen after electrophoresis on ethidium bromide stained 2% agarose gels. For real time RT-PCR SYBR green and platinum Taq polymerase (Invitrogen) were used and the amplifications were performed utilizing the Bio-Rad iQ5 multicolor Real time PCR detection system.

#### The respective primers were

##### CDKN1A

5’-CAGAGGAGGCGCCAAGACAG-3’ (forward) and 5’-CCTGACGGCGGAAAACGC-3’ (reverse); *AUF1*: 5’-GATCAAGGGGTTTTGGCTTT-3’ (forward) and 5’-GTTGTCCATGGGGACCTCTA-3’ (reverse); *CDKN2A*: 5’-CAACGCACCGAATAGTTACG-3’ (forward) and 5’-CAGCTCCTCAGCCAGGTC-3’ (reverse). β-actin: 5’-CCCAGCACAATGAAGATCAAGATCAT-3’ (forward) and 5’-ATCTGCTGGAAGGTGGACAGCGA-3’ (reverse); GAPDH: 5’-GAGTCCACTGGCGTCTTC-3’ (forward) and 5’-GGGGTGCTAAGCAGTTGGT-3’ (reverse);

### Analysis of mRNA stability

Cells were challenged with 5 µg/ml Actinomycin D for various periods of time (0-6 hrs). Total RNA was purified, treated by DNase, and then cDNA was synthesized and used for qPCR. Standard curves for the *CDKN1A* gene were generated to determine the relative concentration of amplified transcripts, and then normalized to GAPDH mRNA level, and the normalized values were used to calculate the half-life of the *CDKN1A* mRNA. One-phase exponential decay curve analysis (GraphPad Prism) was used to assess mRNA decay kinetics.

### Immunoprecipitation and quantitative RT-PCR

Cell lysates were prepared from confluent cells, and 3 mg were incubated in the lysis buffer (50 mM Tris (pH 8), 100 mM NaCl, 10% glycerol, 1× protease inhibitors, 5 mM DTT and 2 U/µL RNasin). Subsequently 5 µg of AUF1 mouse monoclonal antibody (mouse IgG1, used as control) was added and mixed at 4°C for 4 h. Next, equal volume of protein A agarose was added per immunoprecipitation and mixed overnight at 4°C. After centrifugation, the resulting pellet was re-suspended in 1 mL TRI reagent used for RNA extraction and quantitative RT–PCR reactions were performed as described above.

### siRNA, shRNA and ORF transfection

*CDKN2A*-shRNA expressed in pRNAT-U6/Neo vector (GenScript Corporation), pSILENCER-*AUF1-*siRNA and control-siRNA plasmids were used to carryout transfections using the human dermal fibroblast nucleofector kit (Amaxa Biosystems) following the protocol recommended by the manufacturer. The transfections with *CDKN2A*-ORF and control plasmids (SA Biosciences, UK) were performed using Lipofectamine 2000 reagent (Invitrogen).

### Transfection and GFP reporter activity assessment

U2OS and EH1 cells were transfected with EGFP reporter bearing either wild-type or mutant *CDKN1A* ARE using lipofectamine 2000 reagent (Invitrogen), and the assessment of the GFP fluorescence was performed as previously described [35].

### Quantification of protein expression level

The expression levels of the immunoblotted proteins were measured using the densitometer (BIO-RAD GS-800 Calibrated Densitometer) as previously described [32].

### Statistical analysis

Statistical analysis was performed by student’s t-test and *p* values of 0.05 and less were considered as statistically significant.

## Results

### p16 modulates p21 expression in human and mouse cells

**Figure 1:**
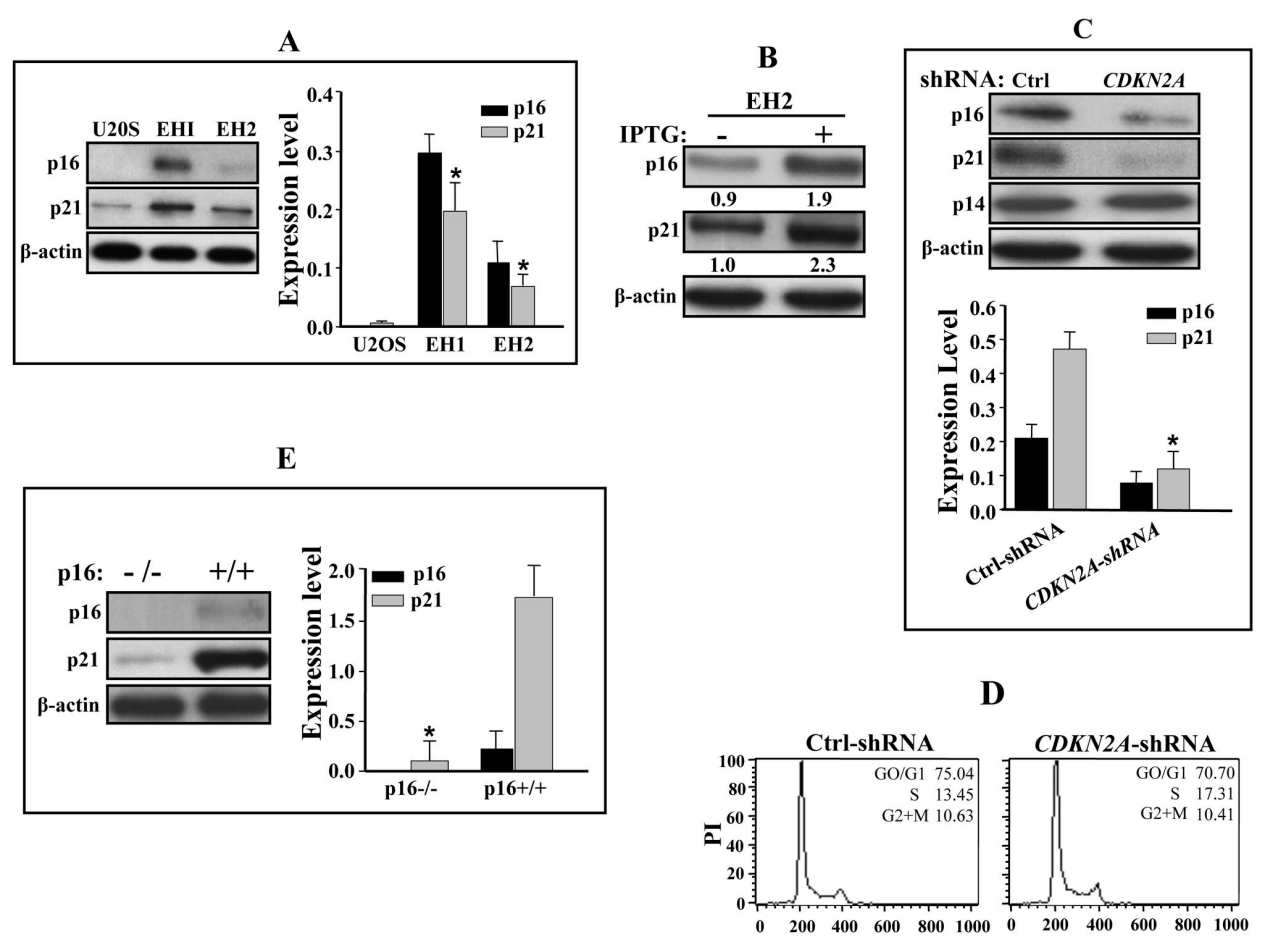
p16 modulates p21 protein level in human and mouse cells. Whole cell extracts were prepared from different human and mouse cell lines and were used for immunoblotting analysis using the indicated antibodies (A, B, C and E). The histograms show the expression levels of the indicated proteins. Error bars indicate standard deviations of 3 different experiments. *: *p*-value < 0.05. (D) Cell cycle analysis of the indicated cells using flow cytometry.

To study the relationship between p16 and p21, we first made use of the p16-defective U2OS cell line and its derivatives EH1 and EH2 that express p16 under the control of an IPTG-inducible promoter [36]. It is noteworthy, however, that in the absence of IPTG, EH1 and EH2 cells express low basal level of p16, comparable to that detected in early-passage human diploid fibroblasts for EH1, but very low in EH2 (Figure 1A). Thus, this system allowed controlled expression of p16 ranging from absent (U2OS), very low (EH2), normal-like (EH1) to higher IPTG-inducible level. Importantly, the p16 expression in these cells does not exert any measurable effect on cellular growth [36]. Therefore, sub-confluent cells with ~70% presenting a 2N DNA content were used to prepare whole cell extracts that were utilized to assess the level of the p21 protein by the immunoblotting technique using specific antibodies, and β-actin was used as internal control. Figure 1A shows that the p21 protein level is modulated in a p16-dependent manner. Indeed, the level of p21 is 6 fold and 20 fold higher in EH2 and EH1 than in U2OS, respectively (Figure 1A, right panel). This level is further higher in EH1 wherein the p16 level is higher than in EH2 (Figure 1A). These results reveal the existence of a link between p16 and the expression of p21. To confirm this, p16 expression was induced using IPTG (1 µM) in EH2 cells, and then the p21 protein level was assessed in both treated and non-treated cells, which have been used when confluent, with more than 80% of cells exhibiting a 2N DNA content (data not shown). Figure 1B shows that the IPTG treatment led to 2 fold increase in the p16 level. Importantly, this increase was accompanied by 2 fold increase in the level of p21 (Figure 1B). This indicates that the increase in the p16 level induces p21 expression, suggesting that the p21 level is positively modulated by p16 in these cells.

Next, we sought to explore the role of p16 in the regulation of p21 expression in normal human skin fibroblasts. To this end, we used HFSN1 cells expressing either *CDKN2A*-shRNA or a scrambled sequence as control. The *CDKN2A*-shRNA was designed to target the p16 specific exon 1α and avoiding the p14^ARF^ message. Indeed, while the p14^ARF^ protein level was not affected, the p16 protein level decreased more than 60% in the *CDKN2A*-shRNA expressing cells as compared to the control cells (Figure 1C). Interestingly, the decrease in p16 level, while did not affect cell proliferation nor cell cycle distribution (Figure 1D), it led to a reduction of 64% in the p21 protein level (Figure 1C, lower panel). This shows that the relationship between these proteins is not restricted to U2OS and its derivatives, but p16 modulates p21 protein level in human skin fibroblasts as well.

To further explore the effect of p16 on the expression of p21, mouse embryonic fibroblasts either p16-proficient (p16+/+) or p16-deficient (p16-/-) were used. Notably, p16 knock out did not affect cell proliferation (Sharpless et al., 2001). Whole cell lysates were prepared from these cells at the same passage (P6), and were used to assess the level of p16 and p21 proteins. Figure 1E shows that the p21 protein level is 10 fold higher in p16-proficient cells than in p16-deleted ones. This result corroborates the results obtained with human cells and shows that p16 regulates the expression of p21 in both human and mouse cells.

### p16 controls the *CDKN1A* mRNA at the post-transcriptional level

**Figure 2:**
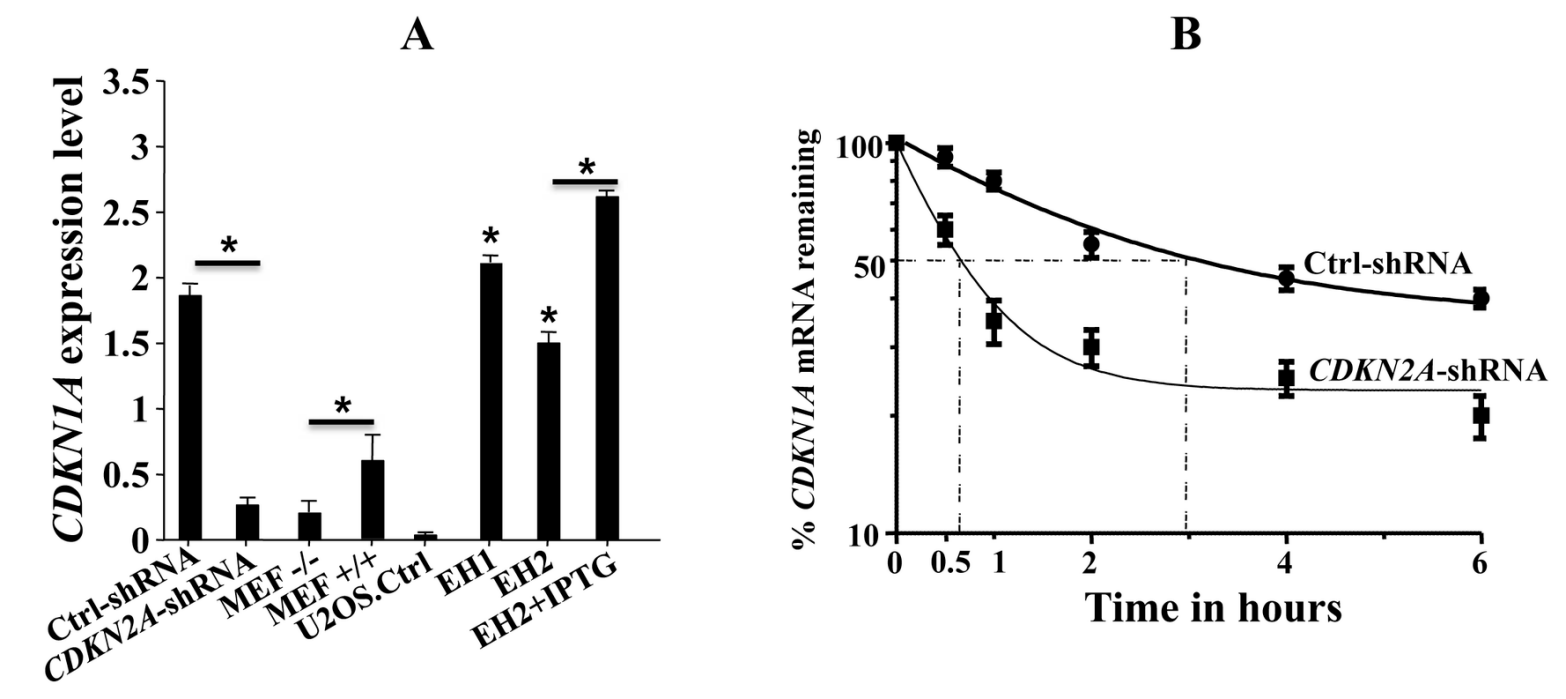
p16 modulates the *CDKN1A* mRNA level in human and mouse cells. (A) Total RNA was extracted from both p16-deficient and p16-proficient cells and used for qRT-PCR utilizing specific primers, and β-actin was used as internal control. Error bars indicate standard deviations of 3 different experiments. *: *p*-value < 0.05. (B) Cells were treated with actinomycin D and re-incubated for different periods of time as indicated. Total RNA was extracted and the *CDKN1A* mRNA level of was assessed by qRT-PCR using specific primers and normalized against β -actin. The graph shows the proportion of the *CDKN1A* mRNA remaining post-treatment, and the dotted lines indicate the *CDKN1A* mRNA half-life. Error bars indicate standard errors of 3 different experiments.

To investigate the mechanism whereby p16 regulates the p21 protein level, we studied the role of p16 in the expression of the *CDKN1A* mRNA in mouse and human fibroblasts. Total RNA was extracted from both p16-deficient and p16-proficient cells and was used for quantitative RT-PCR (qRT-PCR) utilizing specific primers. Figure 2A shows that the *CDKN1A* mRNA level decreased in *CDKN2A*-shRNA expressing cells to a level 83% lower as compared to that in their control counterparts. Similarly, the *CDKN1A* mRNA level was 3 fold higher in p16+/+ cells than in p16-/-MEFs (Figure 2A). In addition, the level of the *CDKN1A* mRNA was 22 fold and 15 fold higher in EH1 and EH2 than in U2OS cells, respectively. The *CDKN1A* mRNA level was further increased upon treatment with IPTG (Figure 2A). These results indicate that the *CDKN1A* mRNA level is modulated in a p16-dependent manner.

Next, we sought to investigate the mechanism whereby p16 modulate the *CDKN1A* mRNA expression. To this end, *CDKN2A*-shRNA expressing cells and their control cells were treated with the transcription inhibitor actinomycin D, and then re-incubated for different periods of time (0-6 hrs). Total RNA was purified and the *CDKN1A* mRNA level was assessed by qRT-PCR. Figure 2B shows that the *CDKN1A* mRNA half-life in the control cells is 3 hrs, while it is only 36 min in *CDKN2A*-shRNA expressing cells. Notably, the kinetics of mRNA decay in *CDKN2A*-shRNA expressing cells showed a biphasic curve, indicating the presence of 2 populations of mRNAs, one was rapidly degraded (up to 2h), while the other one exhibited slow decay similar to that observed in control cells. This may suggest the possible loss of *CDKN2A*-shRNA from a small population of cells, wherein the *CDKN1A* mRNA turn-over was slow. These results show that p16 plays a major role in the stability of the *CDKN1A* mRNA in normal human skin fibroblast cells**.**

### p16 controls the turn-over of the *CDKN1A* mRNA through AUF1

**Figure 3:**
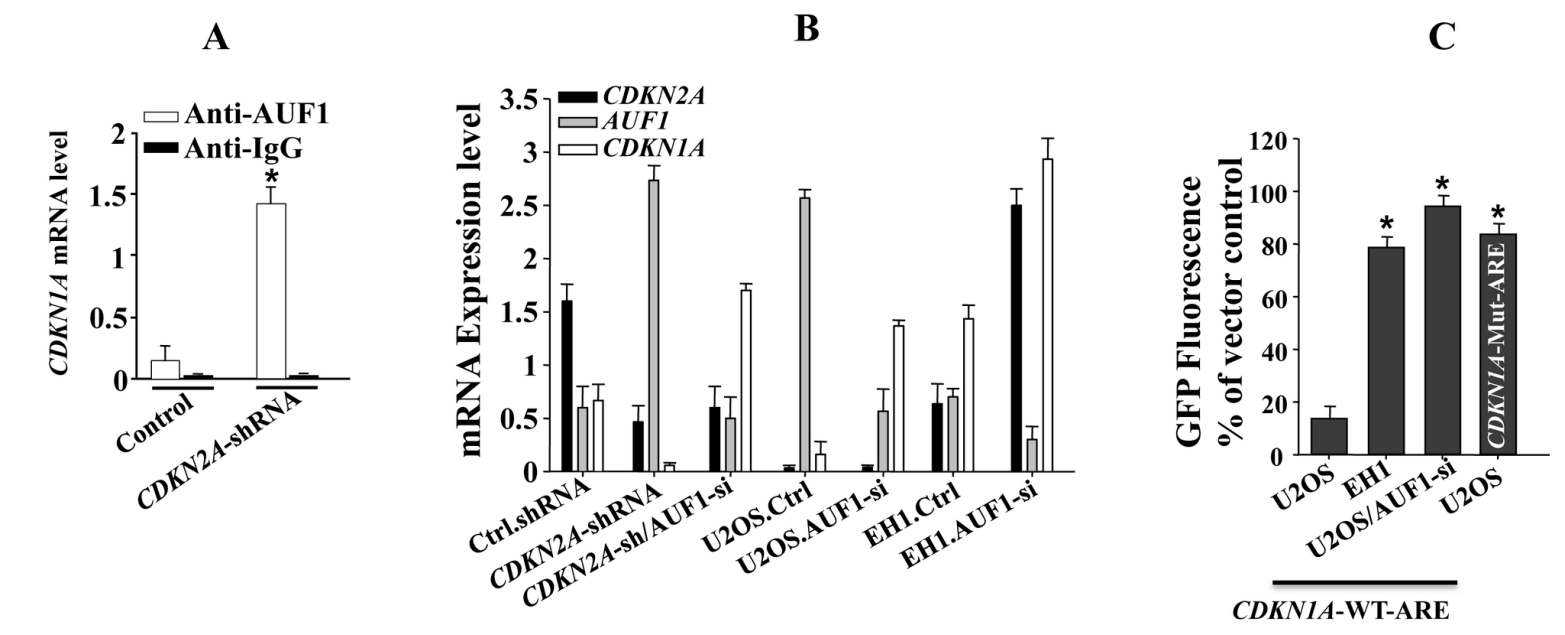
p16 controls the transcription of the *CDKN1A* mRNA through AUF1. (A) Cell lysates were prepared from *CDKN2A*-shRNA expressing cells and their control counterparts and AUF1 mouse monoclonal antibody and mouse IgG (used as control) were utilized for immunoprecipitation. Co-precipitated RNA was purified and used for quantitative RT-PCR reactions. (B) Total RNA was purified from p16 proficient (HFSN1 and EH1) and p16-deficient (U2OS and HFSN1 expressing *CDKN2A*-shRNA) cells, expressing either AUF1-siRNA or control-siRNA. Transcripts for the indicated genes were amplified by quantitative RT-PCR. (C) U2OS cells were transfected with EGFP reporter containing either the wild-type (WT) or the mutated (Mut) *CDKN1A* ARE, while U2OS containing AUF1-siRNA as well as EH1 cells were transfected with the *CDKN1A*-WT-ARE construct. GFP fluorescence was measured 24 hrs post-transfection. Error bars represent means ± S.D. *: *p*-value < 0.05.

Since AUF1 is an RNA binding protein, which destabilizes the *CDKN2A* mRNA [30], we studied the effect of p16 on the binding of AUF1 to the *CDKN1A* mRNA using HFSN1 cells expressing either *CDKN2A*-shRNA or control-shRNA. AUF1-mRNAs ribonucleoprotein complexes were obtained by immuoprecipitation (IP) using anti-AUF1 antibody, and were used for quantitative RT-PCR amplification utilizing specific primers. Figure 3A shows significant increase in the level of the *CDKN1A* mRNAs that were bound to AUF1 in *CDKN2A*-shRNA expressing cells than in control cells, indicating that AUF1 binds to the *CDKN1A* mRNA in a p16-dependent manner. This shows that AUF1 plays a major role in the p16-dependent regulation of p21 expression.

To further elucidate this role of AUF1, we performed double knock-down (p16 and *AUF1*) experiment using specific AUF1-siRNA in HFSN1 cells. Total RNA was purified and amplified by quantitative RT-PCR using specific primers. Figure 3B shows that while the expression level of the *CDKN1A* mRNA decreased in the p16-deficient cells as compared to the control cells, its expression became normal when *AUF1* was down-regulated in p16-defective cells. Similarly, when *AUF1* was knocked-down in the p16-defective U2OS cells, the expression of the *CDKN1A* mRNA increased to a level similar to that observed in the p16-proficient EH1 cells (Figure 3B). Together, these data strongly suggest that p16 positively controls the expression of the *CDKN1A* mRNA through negative regulation of the AUF1 protein.

### p16 regulation of the *CDKN1A* mRNA decay is AU-rich element (ARE)-dependent

To further elucidate the role of the ARE-binding AUF1 protein in the p16-related control of the *CDKN1A* mRNA decay, we utilized EGFP reporter wherein the reporter-coding region was fused with a 70-nt fragment that spans the putative ARE of the *CDKN1A* mRNA. In addition, a mutated putative ARE and a 70-nt of human growth hormone GH1 (non-ARE sequence) were used as controls, as previously described [35]. These constructs were introduced into p16-proficient (EH1) and deficient (U2OS) cells. Figure 3C shows 75% reduction in the GFP level in U2OS cells as compared to EH1 cells, confirming the role of p16 in the stability of the *CDKN1A* ARE-containing 3’-UTR. This reduction was not observed when mutated *CDKN1A* ARE was used (Figure 3C). Interestingly, when AUF1 was down-regulated in U2OS cells, the level of GFP was restored to a level similar to that observed in EH1 cells (Figure 3C). These results show the importance of AUF1 and the *CDKN1A* ARE in p16-dependent control of the *CDKN1A* mRNA decay.

### p16 positively controls p21 expression in breast epithelial cells

**Figure 4:**
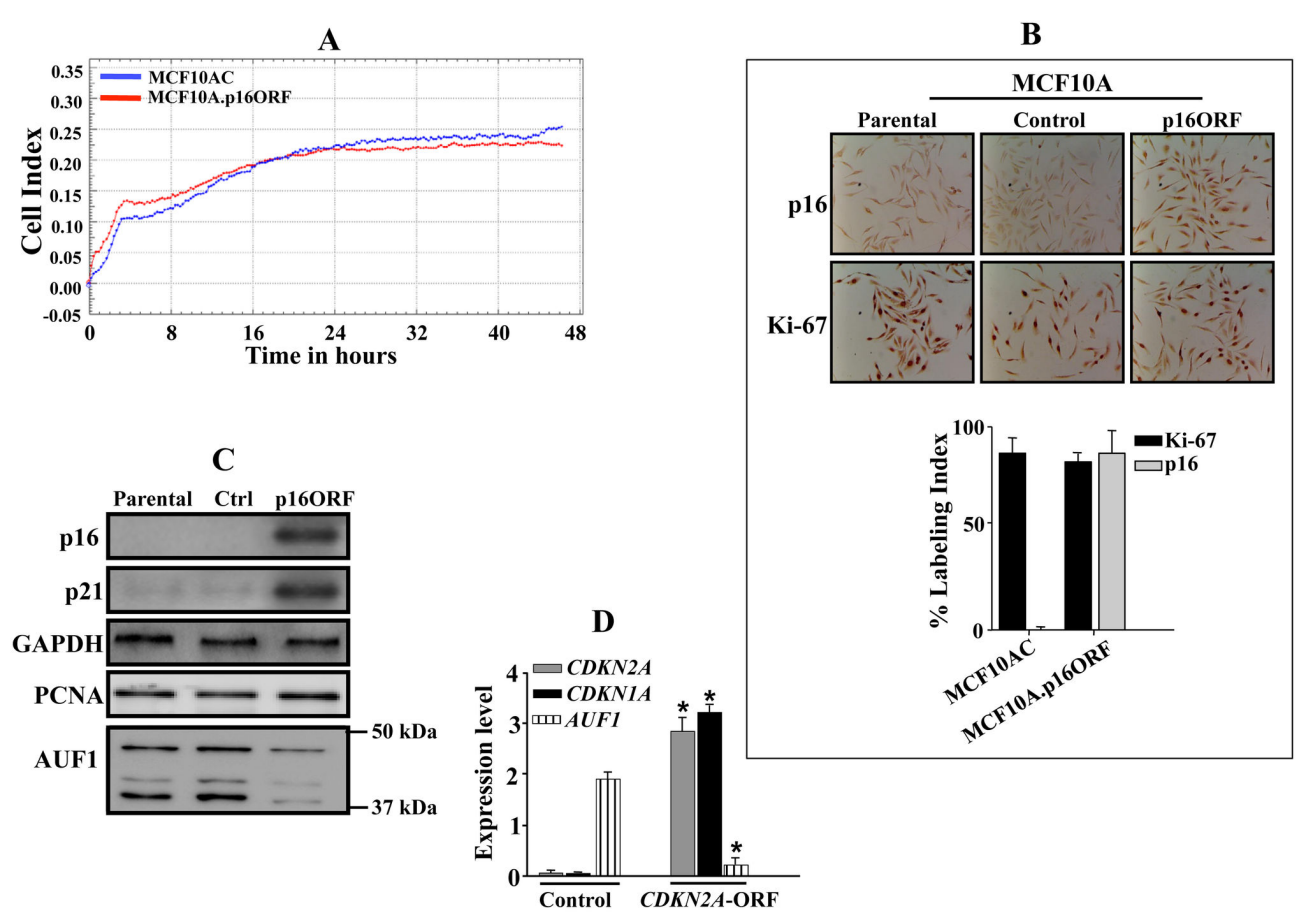
p16 positively controls p21 expression in epithelial cells. MCF-10A cells were transfected with a plasmid bearing the *CDKN2A*-ORF or a control plasmid. (A) 2x10^4^ cells were plated in E-16 plate for 48 hrs and cell proliferation was monitored using the RTCA-DP system. Cell index (CI) represents cell impedance measurement, which represents quantitative information about cell number. (B) p16 and Ki-67 immunostaining. Upper panel: phase contrast microscopy, lower panel: Labeling Index for Ki-67 and p16 staining was determined for at least 500 cells per data point and expressed as mean ± S.D of triplicate determinations. (C) Whole cell lysates were prepared from the indicated cells and were used for immunoblotting utilizing antibodies against the indicated proteins. (D) Total RNA was purified and used for qRT-PCR amplification using specific primers for the indicated genes.

To investigate the link between p16 and p21 in epithelial cells, p16 was ectopically expressed in the p16-defective MCF-10A breast epithelial non-cancerous cells, which express low level of p21. Figure 4A shows that *CDKN2A*-ORF-expressing cells and their control counterparts proliferate similarly. This has been further confirmed by showing that both exhibit similar Ki-67 labeling index (Figure 4B). Next, whole cell lysates and total RNA were prepared from parental cells and *CDKN2A*-ORF expressing cells as well as their control counterparts. Figure 4C shows that expression of p16 in MCF-10A cells significantly increased the level of the p21 protein as compared to its level in the control cells. On the other hand, the AUF1 protein level decreased and the expression of the proliferation marker PCNA remained constant. Figure 4D shows that the expression of p16 in MCF-10A cells decreased the level of the AUF1 mRNA, while increased the level of the *CDKN1A* mRNA. This shows the p16-dependent positive regulation of p21 expression in human epithelial cells as well.

### p16 and p21 levels are positively correlated in various cancer cell lines

**Figure 5:**
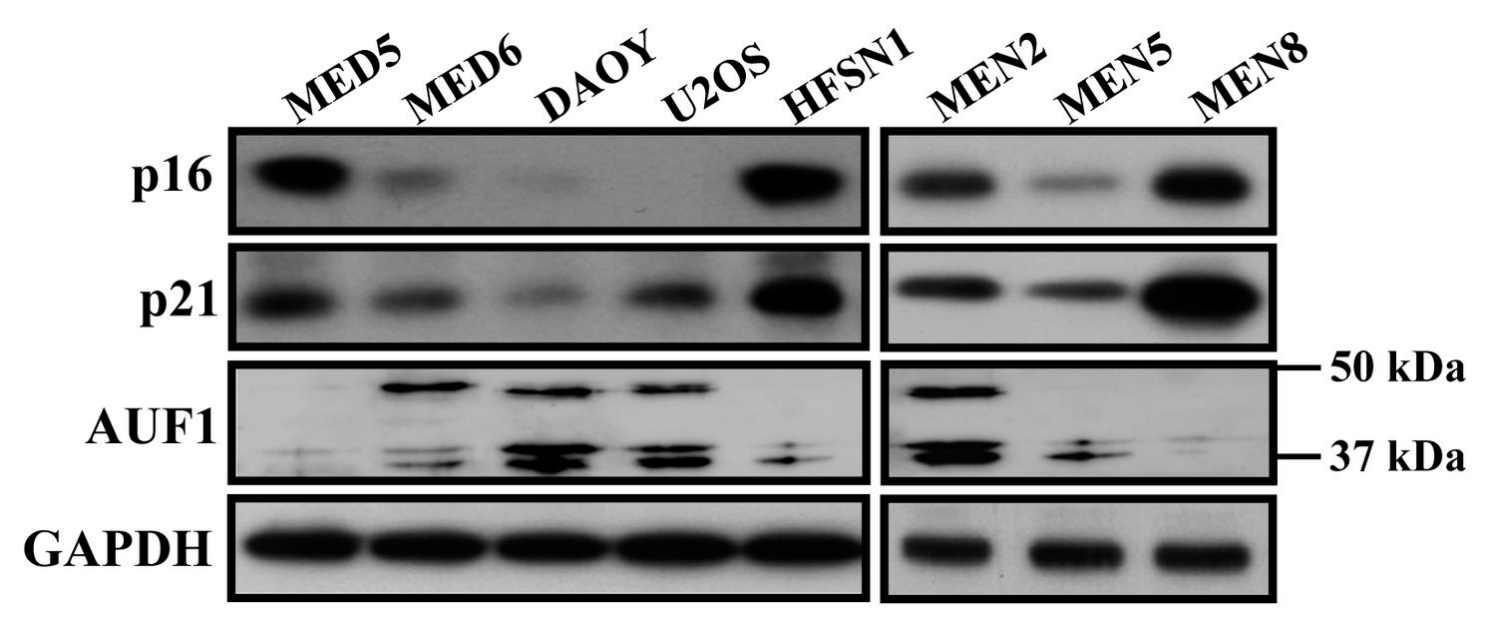
p21 and p16 protein levels are correlative in cancer cells. Whole cell extracts were prepared from the indicated cells and used to assess the levels of p16, AUF1 and p21 by immunoblotting.

Next, we studied the effect of p16 on the expression of p21 in a panel of cancer cells. To this end, protein extracts were prepared from medulloblastoma (MED5, MED6 and DAOY), U2OS, HFSN1 and three meningioma cells (MEN2, MEN5 and MEN8) cells. Figure 5 shows clear positive correlation between the levels of p16 and p21 proteins in these cells. Importantly, the levels of AUF1 negatively correlated with those of p21 and p16, confirming the AUF1-dependent positive regulation of p21 by p16 in various types of cells.

## Discussion

The p14/p53 and p16/pRB pathways are inactivated in most, if not all, human cancers. This shows the importance of these 2 pathways in repressing the carcinogenesis process [18]. p21 is a common element of both pathways and may constitute a bridge of cooperation between them. Indeed, while p21 is the p53 effector during the cellular response to γ-rays, its activity is under the control of p16 in response to UV light [32]. In absence of cellular stresses, p21 expression is tightly regulated by several tumor suppressor genes and oncogenes that belong to these two pathways. These genes receive various intra- and extra-cellular signals, and respond by activating or inhibiting p21, which seems to represent a nodal point for signals from various factors with different roles in carcinogenesis [27,28,37].

In the present report we’ve shown that p16 positively regulates the basal expression of p21 in various human cell types and mouse embryonic fibroblasts. Likewise, using different p16 constructs and the U2OS cell line, it has been previously found that p16 enhances p21 expression [19]. However, in another study, McConnell et al. have found that p16 down-regulates p21 [36]. The reason of this discrepancy is unclear, however, using MEF cells (p16-/-, p16+/+), p16 activation with IPTG and specific *CDKN2A*-shRNA and also ectopic expression of p16 in epithelial cells, we have demonstrated here that p16 is indeed a positive regulator of p21 in mammalian fibroblast and epithelial cells. In addition, we have shown that p16 and p21 protein levels concomitantly fluctuate in various cancer cell lines, which corroborates our findings (Figure 5). Furthermore, we have recently shown that p21 and p16 are both concomitantly down-regulated in various breast cancer-associated fibroblasts as compared to their adjacent counterparts [25,38]. Similarly, a great correlation between p16 and p21 expression has been previously shown in hepatocellular carcinoma. p16 and p21 levels were reduced in these cells in proportion to the degree of methylation of the *CDKN2A* gene [39].

In addition, we have shown that p16 stabilizes the *CDKN1A* mRNA through suppressing the expression of the RNA decay-promoting protein AUF1, known to destabilize the *CDKN1A* mRNA through binding its ARE presents in the 3'-UTR region [30]. Indeed, the *CDKN1A* mRNA turn-over was shown to be p16-related and the p16-dependent modulations in AUF1 levels correlated well with inverse changes in the *CDKN1A* mRNA levels. Moreover, we have shown that AUF1 binds to the *CDKN1A* mRNA in a p16-dependent manner and the decrease in the expression of the *CDKN1A* mRNA in p16-defective cells was restored to normal level by down-regulating *AUF1*, which indicated the capital role of this RNA binding protein in mediating the p16 signaling to p21. This has been confirmed *in vivo* using EGFP reporter bearing *CDKN1A*-ARE. Indeed, these data demonstrated the role of AUF1 and its ARE binding site in the p16-dependent stabilization of the *CDKN1A* mRNA (Figure 3C). In addition to this post-transcriptional regulation of p21, p16 could also control p21 expression at the transcriptional level through AUF1-dependent modulation of transcription factors that control p21 expression. Indeed, AUF1 targets also p53 and C-Myc, which are well known modulators of p21 transcription [30,40,41]. Therefore, p16 may regulate p21 at both transcriptional and post-transcriptional levels in an AUF-dependent manner.

Importantly this link between p16 and p21 is not restricted to mammalian fibroblasts but was also shown in human mammary epithelial cells (MCF-10A). Indeed, ectopically expressed p16 in these p16-defective cells reduced the expression of AUF1 and increased the level of p21 at the mRNA and protein levels. Similar results were obtained when p16 was expressed in breast cancer MDA-MB-231 cells (data not shown).

It is noteworthy that the modulations in the level of p16 that have been used to study the effect on the expression of p21 had only slight effects on cell proliferation. This has been previously shown for U2OS and EH1 cells [36], and also for MEF p16-/- and p16+/+ [42]. Furthermore, p16 knock-down in HFSN1 cells had no effect on the level of the cell cycle regulated HuR protein and also on the cell proliferation PCNA protein [43]. Likewise, ectopic expression of p16 in epithelial cells (MCF-10A) had only slight effects on cell proliferation and the expression of PCNA and Ki-67 (Figure 4). Together, these results rule out the implication of the cell cycle, and indicate that the modulation in the expression of *CDKN1A* is p16-dependent*.*
